# Thalia: semantic search engine for biomedical abstracts

**DOI:** 10.1093/bioinformatics/bty871

**Published:** 2018-10-17

**Authors:** Axel J Soto, Piotr Przybyła, Sophia Ananiadou

**Affiliations:** National Centre for Text Mining, School of Computer Science, University of Manchester, Manchester, UK

## Abstract

**Summary:**

Although the publication rate of the biomedical literature has been growing steadily during the last decades, the accessibility of pertinent research publications for biologist and medical practitioners remains a challenge. This article describes Thalia, which is a semantic search engine that can recognize eight different types of concepts occurring in biomedical abstracts. Thalia is available via a web-based interface or a RESTful API. A key aspect of our search engine is that it is updated from PubMed on a daily basis. We describe here the main building blocks of our tool as well as an evaluation of the retrieval capabilities of Thalia in the context of a precision medicine dataset.

**Availability and implementation:**

Thalia is available at http://nactem.ac.uk/Thalia_BI/.

**Supplementary information:**

Supplementary data are available at *Bioinformatics* online.

The volume, variety and rate of publication for the biomedical scientific literature make it an exemplary case of Big Data and of its inherent challenges. In this information overload scenario, the accurate retrieval of relevant information from such a large volume of written knowledge becomes a necessary asset for biologists and medical practitioners alike (Gonzalez *et al.*, 2016).

In this article, we present Thalia—Text mining for Highlighting, Aggregating and Linking Information in Articles—which is a semantic search tool for the biomedical literature. Its semantic capacity originates from the automatic mining of concepts occurring in articles indexed in PubMed (NCBI Resource Coordinators, 2017) and its normalization to specialized ontologies. In this way, it is possible to search and retrieve all documents containing any mentions of a given concept regardless of the textual variation that is used to represent that concept. Similarly, polysemy—i.e. a same term having multiple meanings—is resolved based on the context where a term occurs. Thalia currently recognizes eight types of concepts, namely: chemicals, diseases, drugs, genes, metabolites, proteins, species and anatomical entities.

Although similar search systems have been made available before (Hoehndorf *et al.*, 2015; Lee *et al.*, 2016; Lu, 2011; Müller *et al.*, 2017; Thomas *et al.*, 2012; Wei *et al.*, 2013), there are several distinctive aspects of Thalia:

1. It is updated daily by automatically downloading updates from PubMed, mining concepts and adding them to the search index. This is a crucial feature, as systems lacking it quickly become outdated after deployment.
2. Thalia’s named entity recognition (NER) methods have been customized for biomedical entity mining as a result of years of research and participation in shared tasks.
3. Thalia uses a context-sensitive acronym resolution in order to improve concept recognition.
4. It provides a visual interface, which allows autocompletion and concept aggregation, as well as a RESTful API that enables programmatic access to the search system.

To recognize named entities from the literature, Thalia uses components of Argo (Rak *et al.*, 2012), which is a text mining workflow system. This includes NER modules for chemicals, drugs and metabolites (Kolluru *et al.*, 2011; Nobata *et al.*, 2011), genes, diseases and proteins (Rak *et al.*, 2014), species (Wang *et al.*, 2010), and anatomical entities (Pyysalo and Ananiadou, 2014). These models are based on dictionary matching as well as conditional random fields models trained using human-annotated data. The recognition step is followed by a normalization (Batista-Navarro *et al.*, 2016) to concepts from the following ontologies: ChEBI (chemicals), DrugBank (drugs), HMDB (metabolites), HGNC (genes), UMLS Metathesaurus (diseases), UniProt (proteins), NCBI (species) and CARO (anatomical).

We leverage our acronym disambiguation module (Okazaki *et al.*, 2010) to improve NER precision and recall. If the long (i.e. spelled out) version of an acronym is recognized as a concept by the NER, but its short form is not, then we can extend the concept on the short form, too. Similarly, if a concept is recognized in an abbreviated form by the NER, but not as the same concept as the one recognized in the long form, then we correct the concept recognition in the short form. This follows the observation that long forms are less ambiguous as NER models can be deceived by *ad hoc* abbreviations.

The search system was implemented using Elasticsearch (https://www.elastic.co/products/elasticsearch), which can be accessed from a web-based interface written in Javascript (Fig. 1). Semantic search is enabled by expanding the query area or by interacting with the different entity facets, which suggest the most frequent entities to narrow down the list of retrieved documents. Thalia also allows inspecting the full text of each abstract with its occurring entities highlighted as well as linking to the concepts in the ontology. Alternatively, the API allows by passing the visual interface to interact with Thalia’s search engine programmatically. The Supplementary Material contains documentation for the web-based interface and the API, as well as a video that shows how users can benefit from the semantic search capacity of Thalia.

**Fig. 1.**
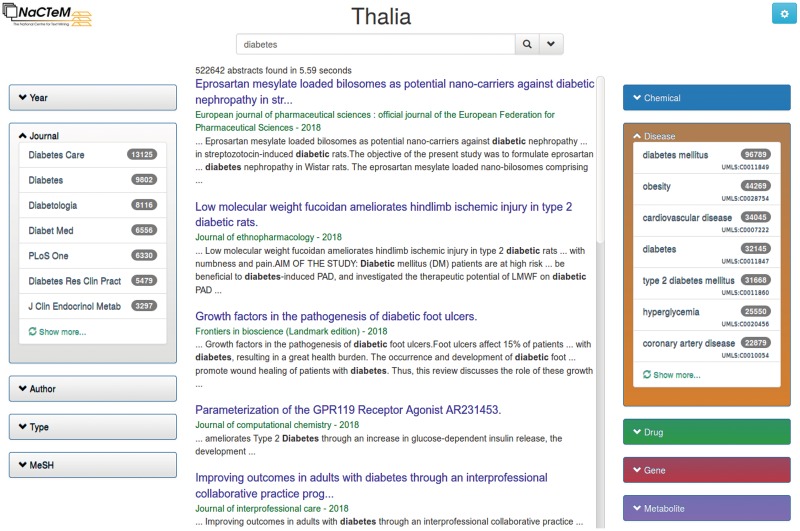
The user interface of Thalia is divided into: a search area (top), main search results pane (middle) and faceted results for publication metadata (left) and entities (right)

We evaluated the search capacity of Thalia in a precision medicine (PM) scenario. In PM, a problem that medical practitioners need to overcome is to find the best treatment given a patient's disease and her genetic features. Herein, we make use of TREC 2017 PM shared task data (Roberts *et al.*, 2017). The challenge involved a set of patient cases, which are described by the patient disease, her genetic variance and other demographic information. The goal was to find documents (PubMed entries, conference abstracts and clinical trials) that are relevant, i.e. they relate to a potential treatment for the patient. Herein, we consider the PubMed part of the task only since this is the corpus that the openly available version of Thalia operates on. More information about the multi-source version could be found in a separate publication (Przybyła *et al.*, 2017).

We experimented using two main search strategies on the TREC PM dataset. The first strategy employed a purely textual search of the disease, gene and demographic data of the patients. Our second strategy incorporated the semantic search capacity of Thalia, which involves textual as well as concept matching. This latter type of matching enables the retrieval of documents regardless of whether the same string occurs in the query and the documents, but depending on whether the same concept is present in the query and the retrieved documents. In this way, vocabulary mismatch between query and documents is addressed, hence improving retrieval performance. The concepts in the query are obtained by using a feature of Thalia that given a term, returns the most likely concept associated with it. The results can be observed from Table 1. As per the shared task evaluation, the results consisted of measuring infNDCG, Precision at 10 and R-prec (Roberts *et al.*, 2017). Note that some of the retrieved documents may have not been assessed by the shared task evaluators, so by taking a conservative approach, those documents were considered as not relevant in this *post hoc* evaluation. This implies that the results in Table 1 represent a lower bound to the actual performance. Additionally, we provide an average time of query processing and retrieval by means of the API. The results indicate that Thalia’s semantic capacity leads to improved retrieval performance with little increase in processing time.

**Table 1.** System performance in terms of infNDCG, precision at 10, R-prec and retrieval time per query in seconds depending on whether the semantic concepts are used for retrieval or not

	infNDCG	P@10	R-prec	Query time
Textual	0.338	0.403	0.213	1.22
Thalia	0.383	0.427	0.230	1.86

## Funding

This work was supported by BBSRC, Enriching Metabolic PATHwaY models with evidence from the literature (EMPATHY) [Grant ID: BB/M006891/1] and The Manchester Molecular Pathology Innovation Centre (MMPathIC) [Grant ID: MR/N00583X/1].

*Conflict of Interest*: none declared.

## Supplementary Material

bty871_Supp
