# Supplementary material for: Thalia: semantic search engine for biomedical abstracts
Source: Bioinformatics. 2018 Oct 17;35(10):1799–801. doi: 10.1093/bioinformatics/bty871 (PMC6513154; doi:10.1093/bioinformatics/bty871)
Supplement: bty871_Supp [file bty871_supp.zip › bty871-Suppl_data/bty871_User_manual.pdf]

Thalia
User manual

Axel J. Soto, Piotr Przybyła, Sophia Ananiadou
National Centre for Text Mining (NaCTeM), UK

Introduction

Thalia (**T**ext mining for **H**ighlighting, **A**ggregating and **L**inking **I**nformation in **A**rticles) is a semantic search engine that can recognise concepts occurring in biomedical abstracts indexed on PubMed (https://www.ncbi.nlm.nih.gov/pubmed/). It currently recognises eight types of concepts, namely: chemicals, diseases, drugs, genes, metabolites, proteins, species and anatomical entities.

This user manual describes the main components and options of the web-based tool as well as it demonstrates the semantic capabilities of Thalia.

The webpage for this project can be found on http://nactem.ac.uk/Thalia (password protected while under review—u: "reviewer", p: "bi"), where further information about the interface as well as any updates on Thalia are posted.

Main interface

Thalia can be accessed using a web browser[1]. Once loaded, after showing a welcome dialog, which also links to this manual, Thalia prompts the user with the initial search interface as shown in Figure 1.

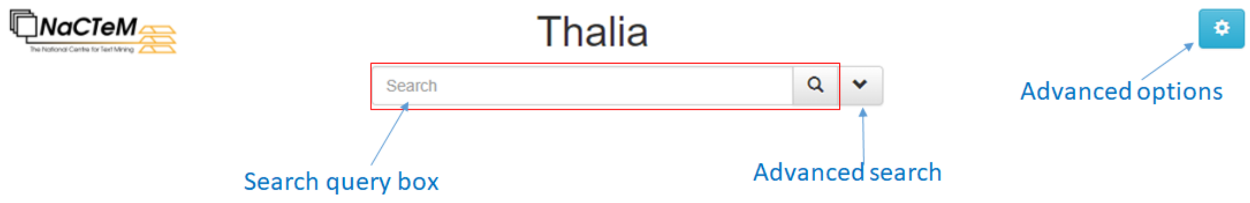

Figure 1: Initial search interface

A text-based search can be executed by typing a query into the search query box and hitting "Enter" key or clicking the magnifying glass icon. For example, if the user enters the text string *GAD*, Thalia will return a list of abstracts from PubMed containing the word *GAD* as shown in

[1] Google Chrome, Safari and Firefox have been tested to be compatible with Thalia.

Figure 2. The middle panel shows a list of the first 20 search results, including the total number of matching documents and the time taken, which are shown at the top. The user can scroll down the middle panel to inspect each of the snippets and click on the bottom arrows to show the next set of 20 results, if needed.

Side panels show two expandable sets of result facets. The facets on the left are related to publication meta-data—i.e. *Publication Year*, *Journal Name*, *Author*, *Publication Type* and *MeSH tags*—while the ones on the right correspond to entity facets—i.e. *Chemical*, *Disease*, *Drug*, *Gene*, *Metabolite*, *Protein*, *Species* and *Anatomical*—. Within each facet there is a list of values with a number indicating the document frequency as a badge. This means that after clicking on a specific item, the next search will be filtered by the documents containing that facet value, and the total number of filtered documents will be the number indicated in the badge. In the case of entities, the facets also show the corresponding concept identifier in an ontology associated with the entity type. Note that the search query box can also parse more complex queries, including phrasal queries—e.g. *"homo sapiens"* (note the quotes)—or boolean queries—e.g. *GAD AND diabetes*.

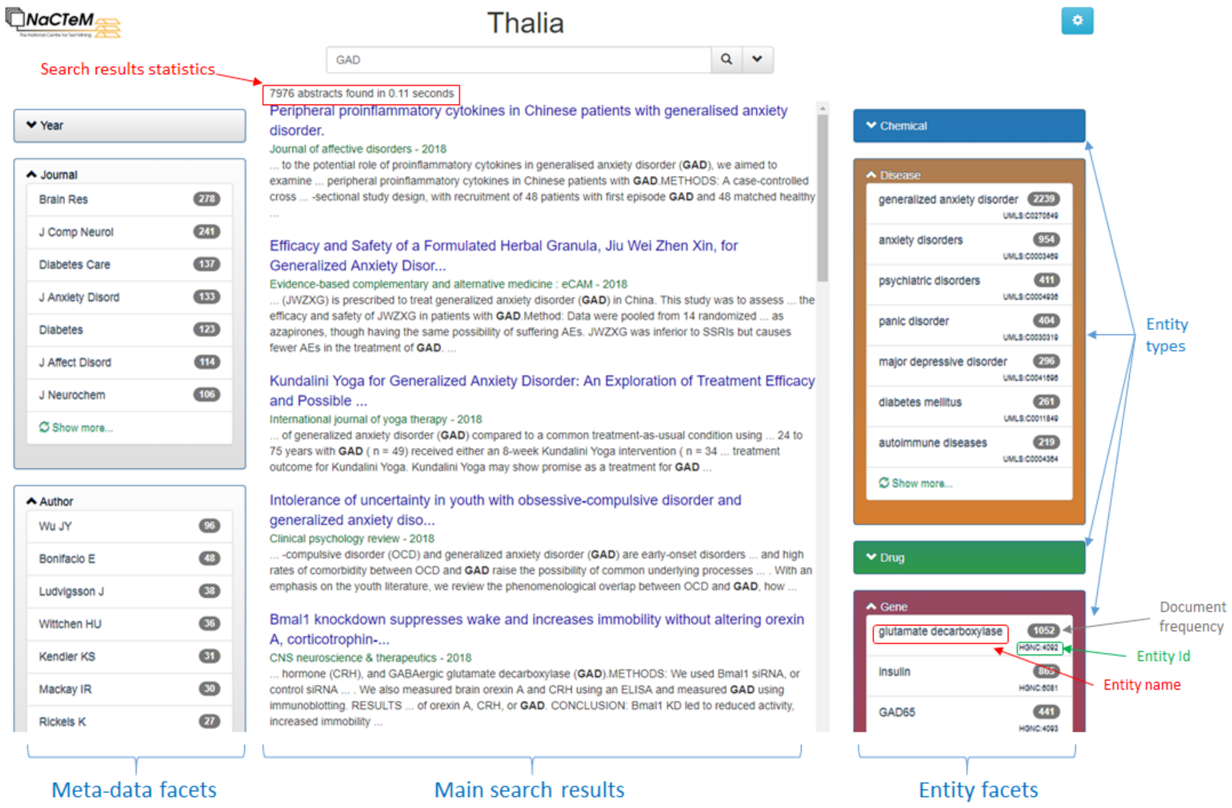

Figure 2: Results for a text-based search

We can observe from the concepts listed in the entity facets in Figure 2 that *GAD* is an ambiguous term that in certain contexts can refer to the disease *generalized anxiety disorder* or the gene *glutamate decarboxylase*. This is where the semantic capacity of Thalia can be used to disambiguate which of the two senses were intended by the user who formulated the query.

Let us assume that the user is interested in the gene. After clicking on *glutamate decarboxylase* from the gene facet, a new search is triggered that enforce the HGNC:4092 concept to be present in the documents retrieved. Although this concept has *glutamate decarboxylase* as its most common name, it has other possible names, such as: *GAD*, *GAD1* and *glutamate decarboxylase 1*, *glutamate decarboxylase 1 (brain, 67kD)* and *glutamate decarboxylase 1 (brain, 67kDa)*. The result of this procedure is shown in Figure 3, where the number of results retrieved is 1052 (down from 7976)[2]. At the same time we can see that the clicked concept has been added to the advanced search panel. This advanced search panel can be visualised by clicking on the caret besides the query search box.

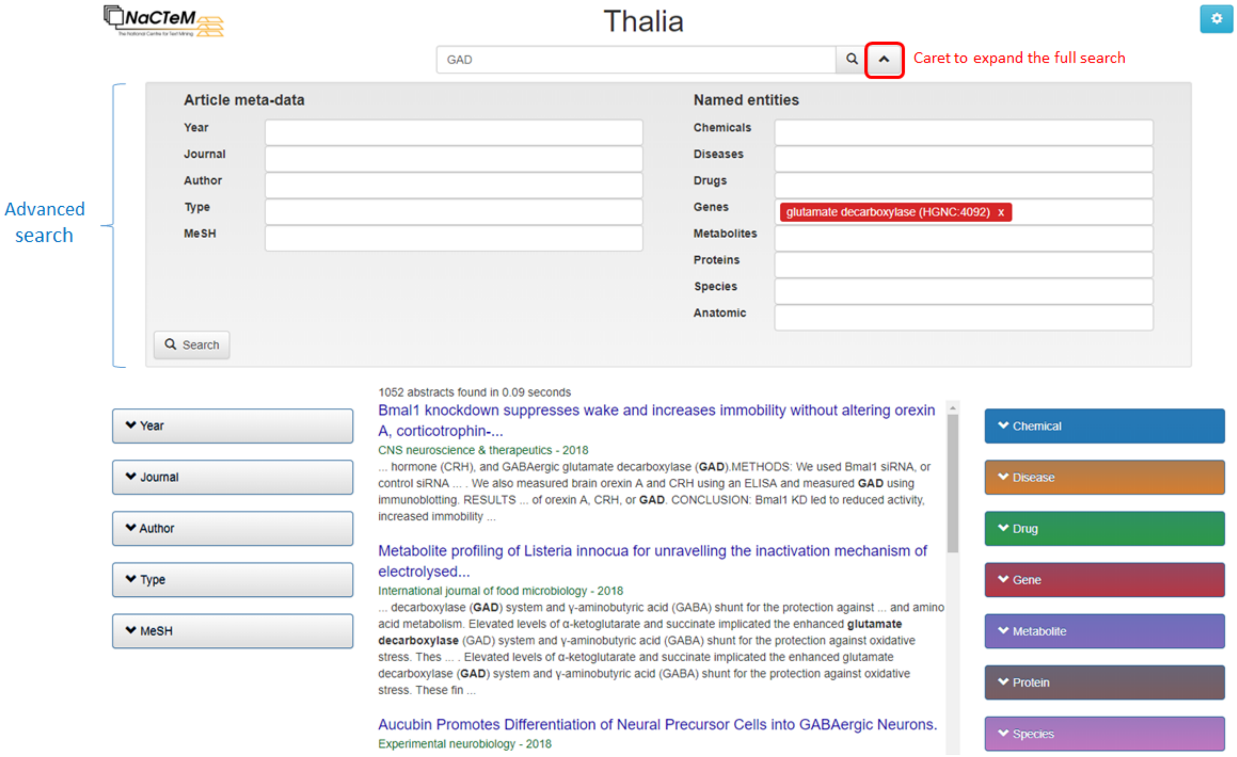

Figure 3: Thalia after clicking on one value of the gene facet

[2] The exact numbers may be different due to the continuous update carried out on Thalia.

The advanced search panel allows to type in article meta-data as well as named entities to include in the search query. To facilitate the accurate specification of the terms in the advanced search, an auto-completion mechanism is in place, as it is shown in Figure 4.

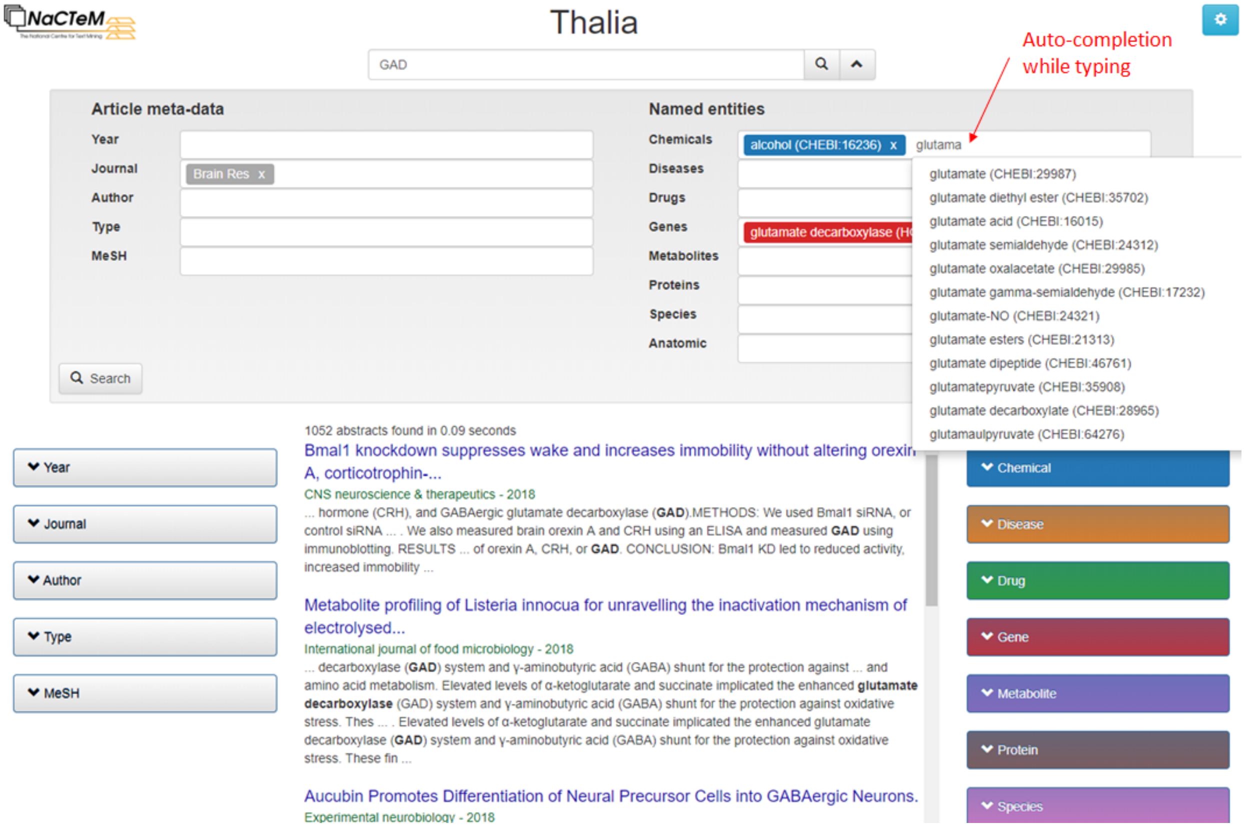

Figure 4: Auto-completion of values in the advanced search panel

Once a value is entered in the advanced search—either typed-in or selected from the facets—, the term is shown enclosed with a tag. In the case of named entities, if normalised, the tag will exhibit the concept identifier in parentheses. As each entity type has a color associated to it, the tag will be color-coded accordingly. In the case of the meta-data values, the tags are always gray. Each of these tags will be interpreted in the query as a conjunction, which means that a boolean AND operation is used to aggregate all the values. In order to use a disjunction, the user has to click on the tag, which will lead to a new window for entering the concepts for disjunction (Figure 5a). Once the desired disjunction is entered, this type of tags is shown using a dark thick border (Figure 5b).

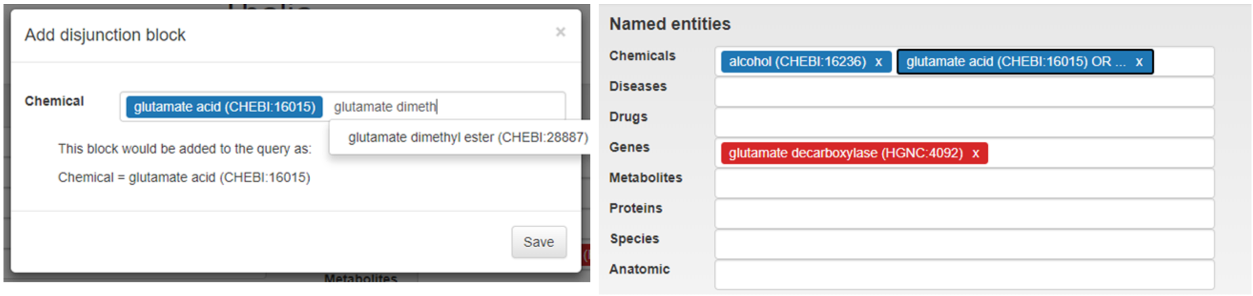

a) b)

Figure 5: Process of adding a term to be used as a disjunction in the query. a) Entering a term to be aggregated using a disjunction. b) The entities that form a disjunction block are shown with a thicker border.

An entity normalised to a concept in an ontology can be inspected by Ctrl-clicking (Command-clicking for Mac users) on the tag, which will open the web page of the ontology concept being clicked. All the ontologies are openly available, except for UMLS (used for diseases), which requires free user registration.

Full text view

Retrieved abstracts can be inspected upon clicking on an entry of the retrieved document list. Thalia will show a window similar to the one in Figure 6. Each recognised entity is highlighted with the color of its corresponding type. Multiple-typed entities are visualised with a black-bordered rectangle. At the bottom of the panel, there are toggle buttons that allow switching on or off the highlighting of entities of a specific type. At the top of the panel there is a light blue button to open a new page with the PubMed entry of that article.

Hovering over a highlighted entity will pop up a label with its concept identifier. When an entity is clicked, the tool will open the ontology web page for the corresponding concept. In the case of entities highlighted as multiple-typed, the user needs to switch off some entity types until just one type is highlighted for the entity, before the user can be redirected to the ontology entry.

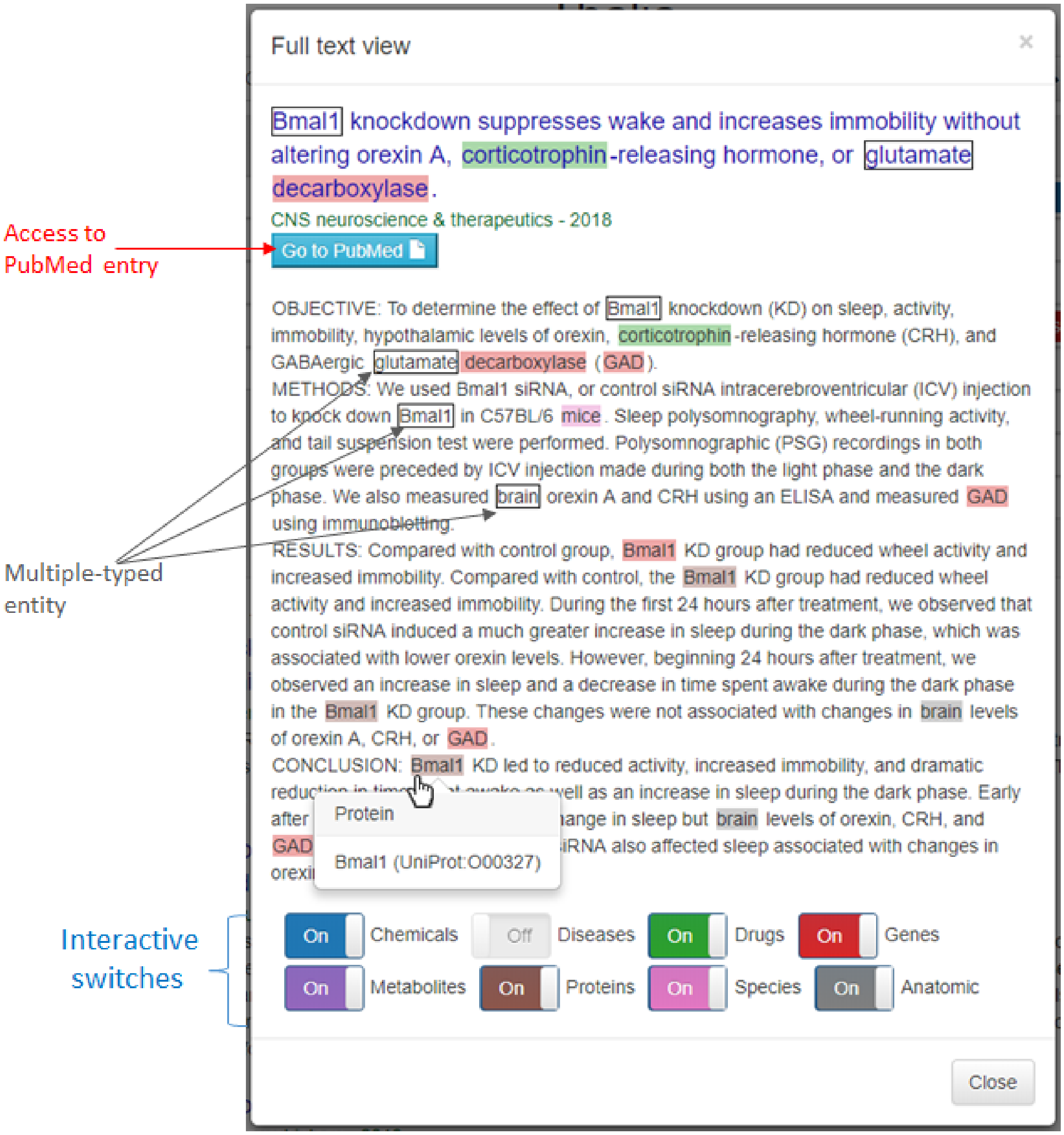

Figure 6: Full-text view

Advanced options

Advanced options for Thalia can be accessed by clicking the light blue gear icon located at the top right of the screen (see Figure 1). There are four main items accessible from this panel as it is shown in Figure 7.

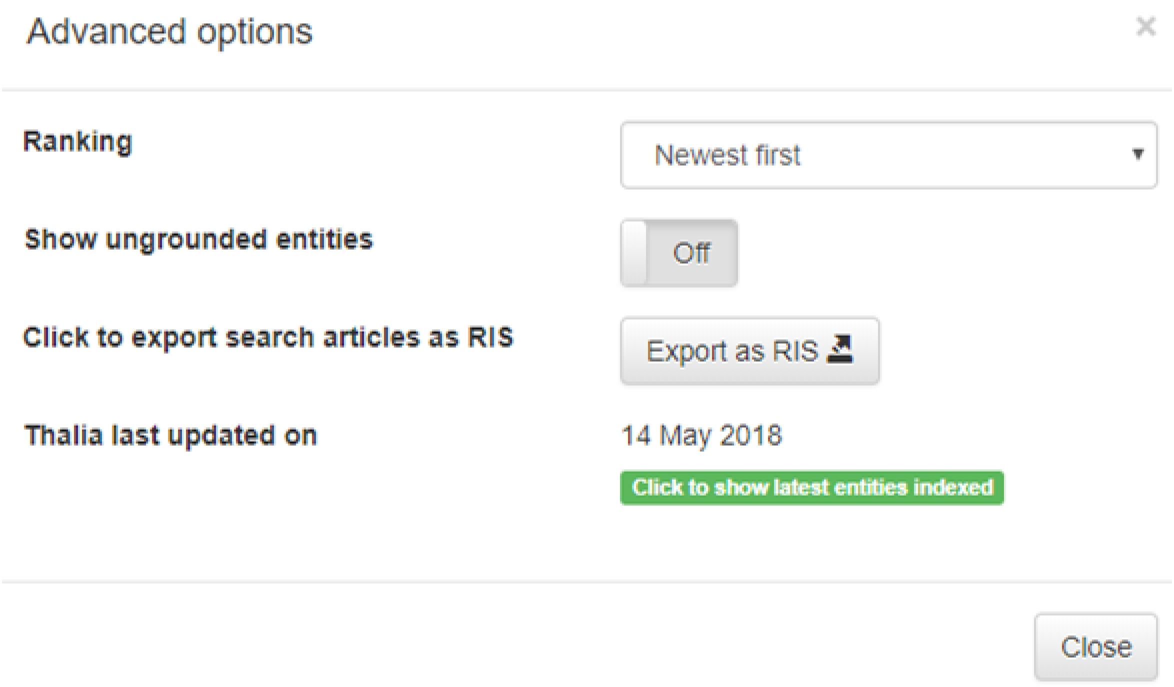

Figure 7: Advanced options

The first option allows to choose ranking retrieved documents either chronologically or by relevance. For generic queries it is usually most convenient to focus on the latest results first. If the query gets more specific, it may be better to rank by relevance. The second option determines whether ungrounded entities are taken into account or not. By default, only entities that could be normalised to concepts are shown to the user (during autocompletion, as facetted values or as highlights in the document view). The user can, however, take into account entities that failed to be grounded by enabling this second option. The third option allows to export the set of retrieved documents as a RIS file (there is a limit currently set to 1000 for the maximum number of documents that can be exported at once). Finally, there is a date that shows when Thalia was last updated. The user can click on this item to show which were the latest entities updated in the form of a word cloud (Figure 8). After choosing between *Last week* or *Last month* and clicking on the *Visualize entities* button, the user can inspect the most frequent entities in the documents added during the chosen period. Similarly to the Full Text View, the user can interact with the switches to turn on or off certain types of entities.

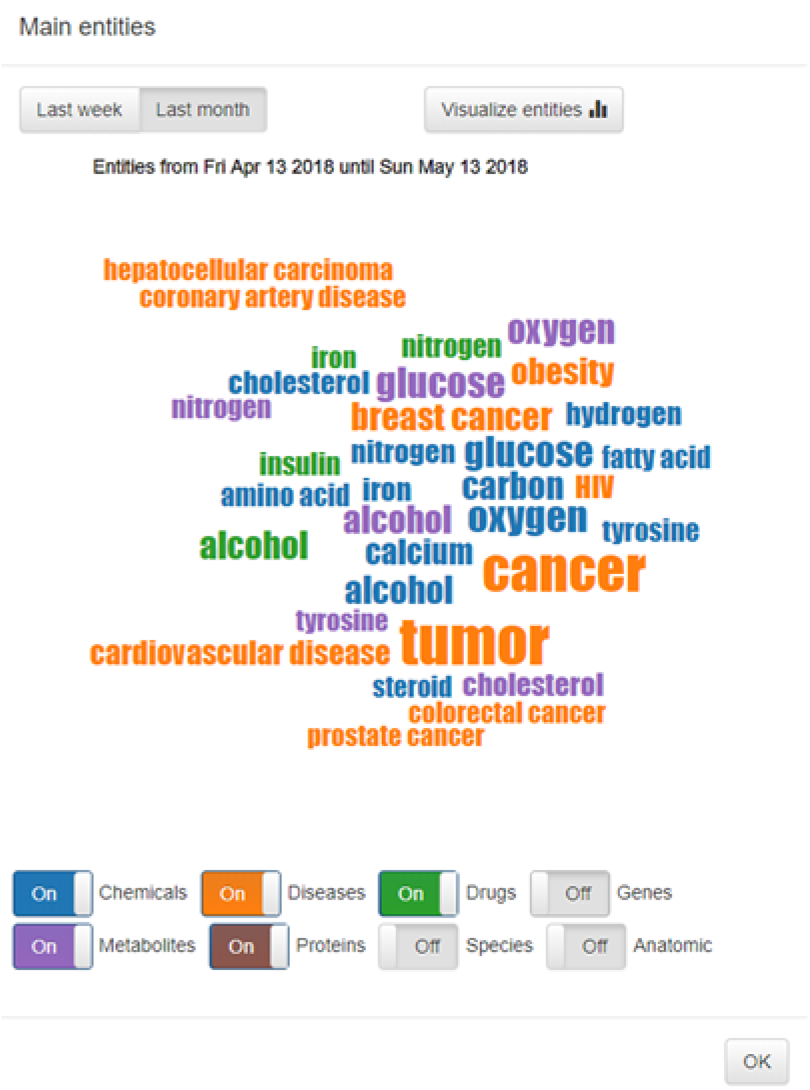

Figure 8: Word cloud with the most frequent entities indexed by Thalia during the last month

Attribution

If you use Thalia API in your research or project, please cite the following article.

Axel J. Soto, Piotr Przybyła, Sophia Ananiadou, "Thalia: Semantic search engine for biomedical Abstracts", Bioinformatics, Under review.
