# Supplementary material for: Thalia: semantic search engine for biomedical abstracts
Source: Bioinformatics. 2018 Oct 17;35(10):1799–801. doi: 10.1093/bioinformatics/bty871 (PMC6513154; doi:10.1093/bioinformatics/bty871)
Supplement: bty871_Supp [file bty871_supp.zip › bty871-Suppl_data/bty871_API_Manual.pdf]

Thalia
API manual

Axel J. Soto, Piotr Przybyła, Sophia Ananiadou
National Centre for Text Mining (NaCTeM), UK

Introduction

Thalia (**T**ext mining for **H**ighlighting, **A**ggregating and **L**inking **I**nformation in **A**rticles) is a semantic search engine that can recognize concepts occurring in biomedical abstracts indexed on PubMed (https://www.ncbi.nlm.nih.gov/pubmed/). It currently recognizes eight types of concepts, namely: chemicals, diseases, drugs, genes, metabolites, proteins, species and anatomical entities.

This manual describes how its RESTful API can be used to query Thalia using HTTP requests. The webpage for this project can be found on http://nactem.ac.uk/Thalia (password protected while under review—u: “reviewer”, p: “bi”), where further information about the API as well as any updates on Thalia are posted.

Instructions

Thalia API can be accessed by sending HTTP requests using the POST method to a single address. The current URL for Thalia API can be found on this webpage:
http://nactem.ac.uk/Thalia/ (password protected while under review—u: “reviewer”, p: “bi”)

The request body follows a JSON format (*Content-Type* header set to *application/json*) with the following structure:

```
{
   "triples": [
      {
         "type": <string>
         "id": [<string>, …, <string>],
         "word_form": [<string>, …, <string>]
      },
      …,
      {...}
   ],
   "from": <integer>,
   "size": <integer>
```

```
}
```

The format uses the same elements as the one used on Thalia's web interface, which is:

- A list of triples that are aggregated by a logical AND in the query. Each of these elements would represent a tag of the advanced search interface (see figure below).

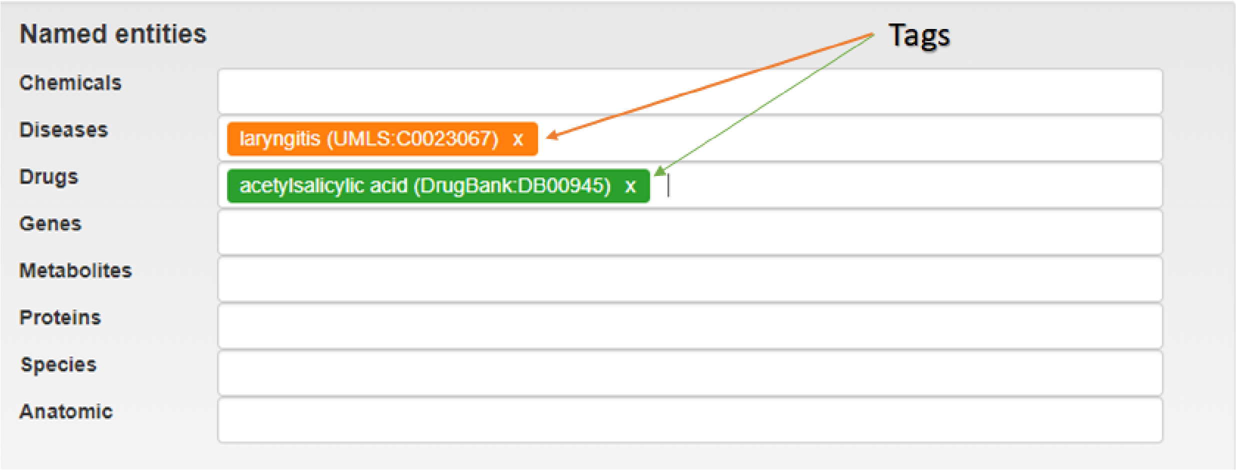

- Type should be one of these values: "Chemical" | "Disease" | "Drug" | "Gene" | "Metabolite" | "Protein" | "Species" | "Anatomical" | "None"
  - "None" is used for text-based search (i.e. this would be analogous to using the main search query box)
- Either the "id" or the "word_form" field should be used. When "id" is used, it should follow the format <Ontology_prefix>:<id_number>. However, in some cases the ontology identifier may not be known, so the "word_form" can be used instead, and Thalia will automatically translate the given word form to the most likely identifier.
- Note that the fields "id" and "word_form" allow to specify a list (i.e. more than one string value). In this case, the values within this list are aggregated using a boolean OR (this would be analogous to the disjunctive block in the web interface).
- The "from" and "size" fields allow pagination by providing the index of the first document retrieved ("from") and the number of documents retrieved ("size").
  - Note that the sum of "from" and "size" cannot be greater than 1000.

For example, this request:

```
{
   "triples": [
      {
         "type": "Chemical",
         "id": ["CHEBI:17234", "CHEBI:16236"],
         "word_form": []
      },
```

```
        {
            "type": "Species",
            "id": [],
            "word_form": ["yeast"]
        }
    ],
    "from": 0,
    "size": 10,
    "boosting": true
}
```

would be translated to: (CHEBI:17234 OR CHEBI:59118) AND (NCBI:4932) and it will return the top 10 results.

Response format

The response is a JSON-based string. The most important object is the one with key “results” that inside has an array “hits”, which in turn contains the details of each returned document.

For example, for the query above, this is the expected response format:

```
{
    "results": {
        "hits": [
            {
                "_score": 25.67723,
                "_source": {
                    "MedlineCitation": {
                        "pmid": {
                            "content": "9784152",
                            "version": "1"
                        }
                    }
                }
            },
            {
                "_score": 18.33628,
                "_source": {
                    "MedlineCitation": {
                        "pmid": {
                            "content": "10791742",
                            "version": "1"
```

```
                }
            }
        }
    },
    …,
    {
        "_score": 18.33628,
        "_source": {
            "MedlineCitation": {
                "pmid": {
                    "content": "9603034",
                    "version": "1"
                }
            }
        }
    }
    ],
    "max_score": 25.67723,
    "total": 11258
  },
  "timed_out": false,
  "took": 73
}
```

Each *hit* contains the objects with keys “_source” and “MedlineCitation” the PubMed identifier (“pmid”) of the article retrieved along with its “version”. Each *hit* also contains the retrieval score. Other statistics-related fields are also part of the response object, such as “max_score” (score of the first hit), “total” (total number of documents matching the query), “timed_out” (whether the query timed out or not), and “took” (time in milliseconds).

Attribution

If you use Thalia API in your research or project, please cite the following article.

Axel J. Soto, Piotr Przybyła, Sophia Ananiadou, “Thalia: Semantic search engine for biomedical Abstracts”, Bioinformatics, Under review.
